# Torsion and Gangrene of a Giant Meckel's Diverticulum in a Middle-Aged Adult: A Case Report

**DOI:** 10.7759/cureus.71457

**Published:** 2024-10-14

**Authors:** Riya B Vakil, Anand Zingade, Saurabh Dumbre, Dattatray Toradmal

**Affiliations:** 1 General Surgery, Pimpri Chinchwad Municipal Corporation's Postgraduate Institute Yashwantrao Chavan Memorial Hospital, Pune, IND

**Keywords:** acute surgical abdomen, giant meckel’s diverticulum, meckel's diverticulum complications, torsion of meckel's diverticulum, true diverticulum, vitello-intestinal duct

## Abstract

Meckel's diverticulum is a true outpouching or diverticulum of the small intestine and is an unusual entity in itself. Typically around 2 inches long, it is usually an incidental finding. Symptomatic patients are mostly children. This is a rare case of a 40-year-old adult Indian male presenting with acute abdomen and found to have torsion and gangrene of a giant Meckel’s diverticulum at emergency laparotomy. The case report aims to highlight the importance of Meckel's diverticulum and its complications as an important differential of an acute abdomen, with the potential for serious morbidity. It serves as a strong reminder to all surgeons to practice routine bowel mapping during all laparoscopic and open abdominal surgeries.

## Introduction

Meckel’s diverticulum is named after Johann Friedrich Meckel, who discovered its origin in 1809 [1,2]. It is an unusual entity, and typically asymptomatic. Following the rule of 2s [3], it has an incidence of approximately 2% [1,2] with only 2% of patients showing symptoms [2], with complications commonly presenting at two years of age [4].

It is a true diverticulum, encompassing all the layers of the gastrointestinal tract (GIT), and the most common congenital malformation of the GIT. Incomplete involution of the vitelline or omphalomesenteric duct, which normally occurs in the fifth to seventh week of fetal development, results in the formation of Meckel’s diverticulum [5]. This diverticulum is usually found 40-50 cm proximal to the ileocecal valve but can be found within 90 cm on average [6]. Usually only 2 cm long, a giant Meckel’s diverticulum (larger than 5 cm) is even rarer and more prone to complications [1,2,7].

Here, we present a rare case of a 40-year-old Indian male patient presenting with acute abdomen, and found to have torsion of a giant Meckel’s diverticulum at an emergency laparotomy.

## Case presentation

A 40-year-old Indian male patient presented to the emergency department complaining of abdominal pain for the past three days. The pain was in the right lower quadrant, and moderate in intensity. There was no history of radiation and no aggravating or relieving factors. There was no history of fever, vomiting, anorexia, distension, or urinary complaints. The patient was passing stools regularly. There was no past medical or surgical history.

On physical examination, the patient was afebrile with vital signs as follows: pulse rate of 130 beats per minute, blood pressure of 80/60 mmHg, respiratory rate of 20 breaths per minute, and saturation on room air of 96%. On per abdominal examination, severe tenderness was elicited in the hypogastrium, right iliac fossa, and right flank.

**Figure 1:**
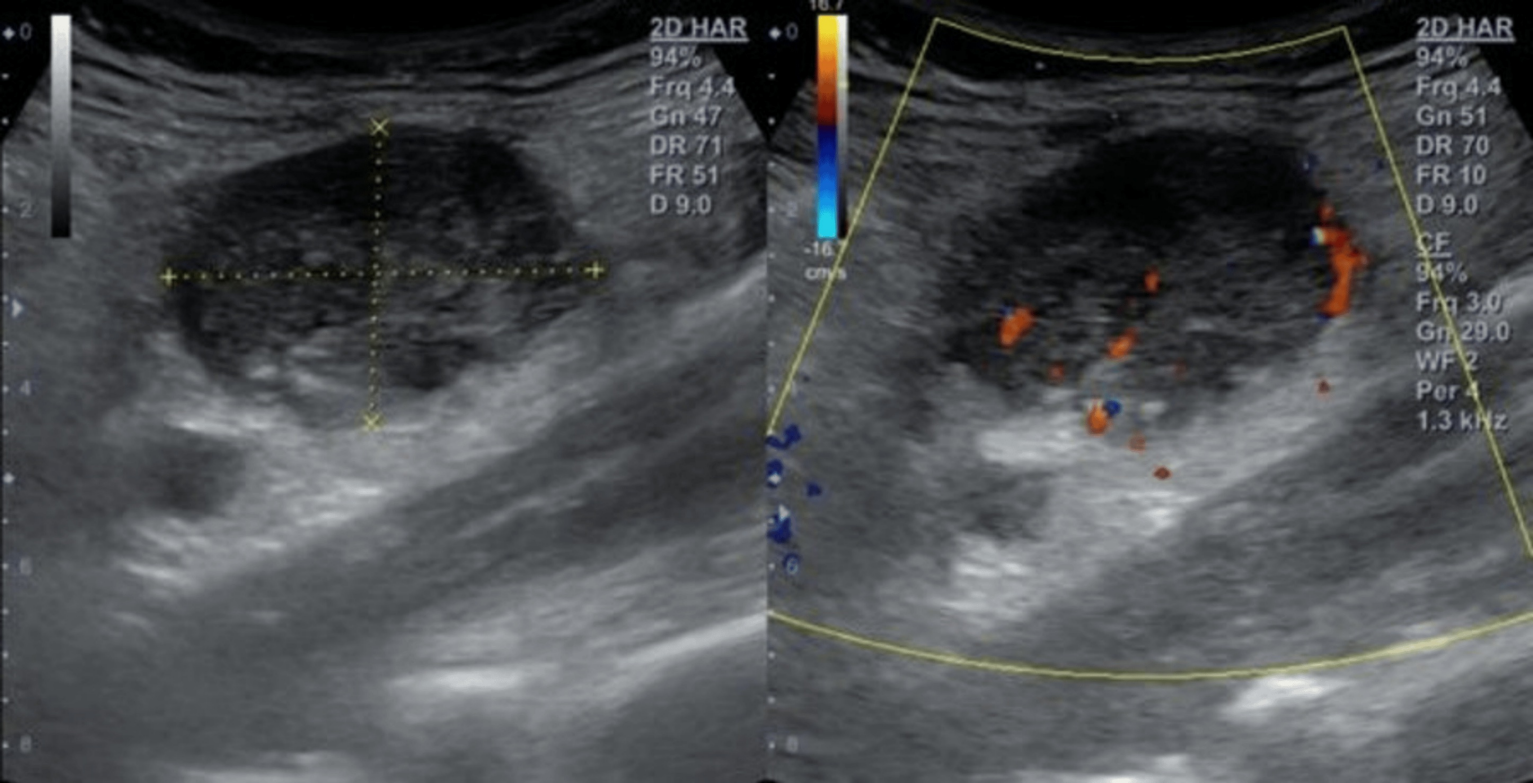
Ultrasonography of the abdomen and pelvis showing a right iliac fossa lump.

An emergency hemogram was run and revealed leukocytosis (WBC count = 16.1 x 10^3^/μl), which consisted of 86% neutrophils, 8% lymphocytes, and 1% eosinophils (Table 1). A plain erect abdominal X-ray was unremarkable. Ultrasonography of the abdomen and pelvis showed only a lump in the right iliac fossa. The appendix was not visualized separately from the lump on ultrasound (Figure 1).

**Table 1:** Relevant findings of the complete hemogram of the patient on admission.

Parameter	Patient Values	Units	Reference Ranges
Hemoglobin	13.7	g/dl	12.0-17.0
RBC count	5.22	mill/cumm	4.5-6.5
WBC count	16100	cells/cumm	4000-11000
Platelet count	3.69	lakh/ul	1.5-4.0
Differential leucocyte count			
Neutrophils	86	%	40-70
Lymphocytes	8	%	20-40
Monocytes	5	%	02-08
Eosinophils	1	%	01-04
Basophils	0	%	0-01

**Figure 2:**
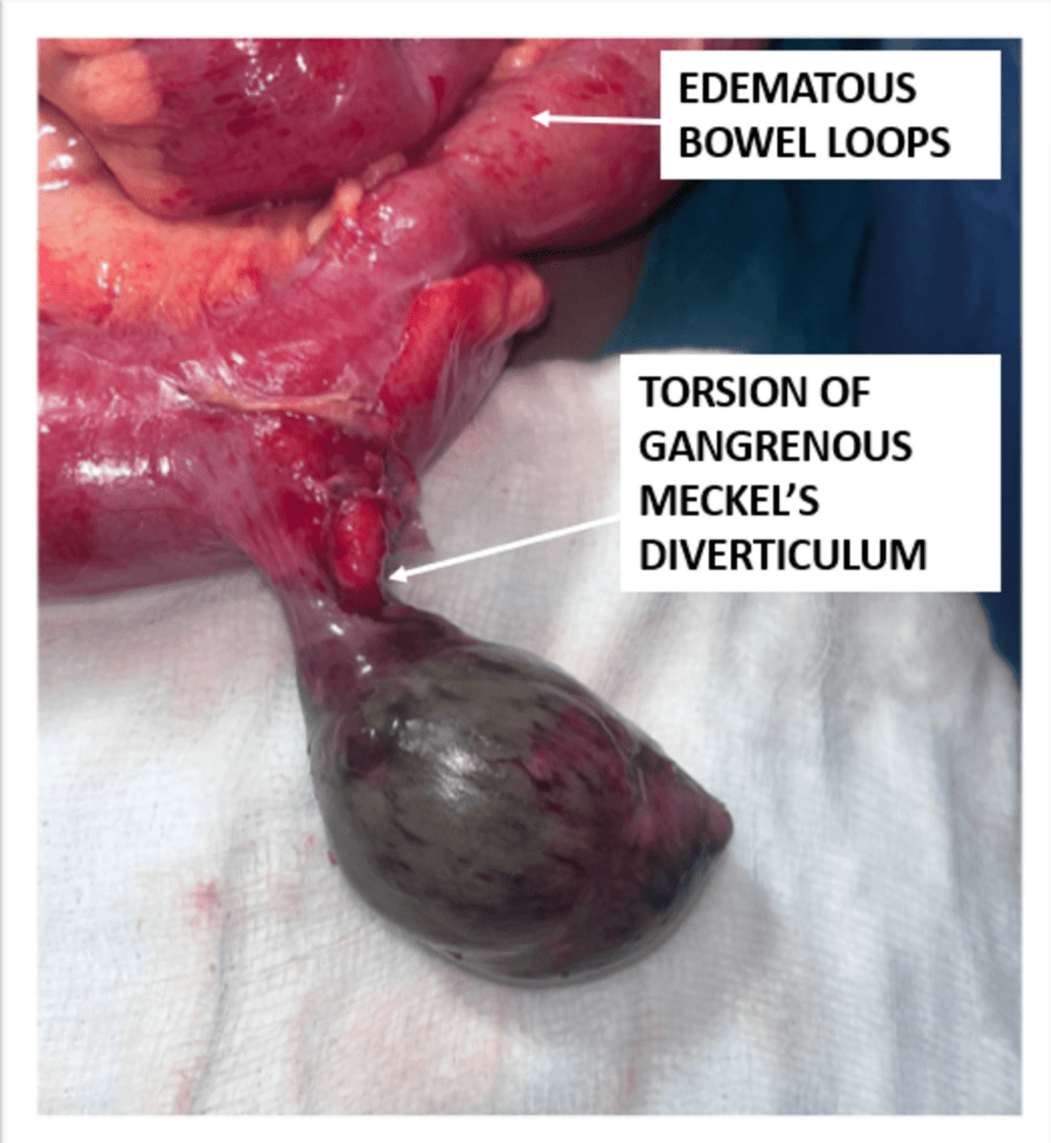
Torsion and gangrene of a giant Meckel’s diverticulum discovered 60 cm proximal to the ileocaecal junction at laparotomy.

**Figure 3:**
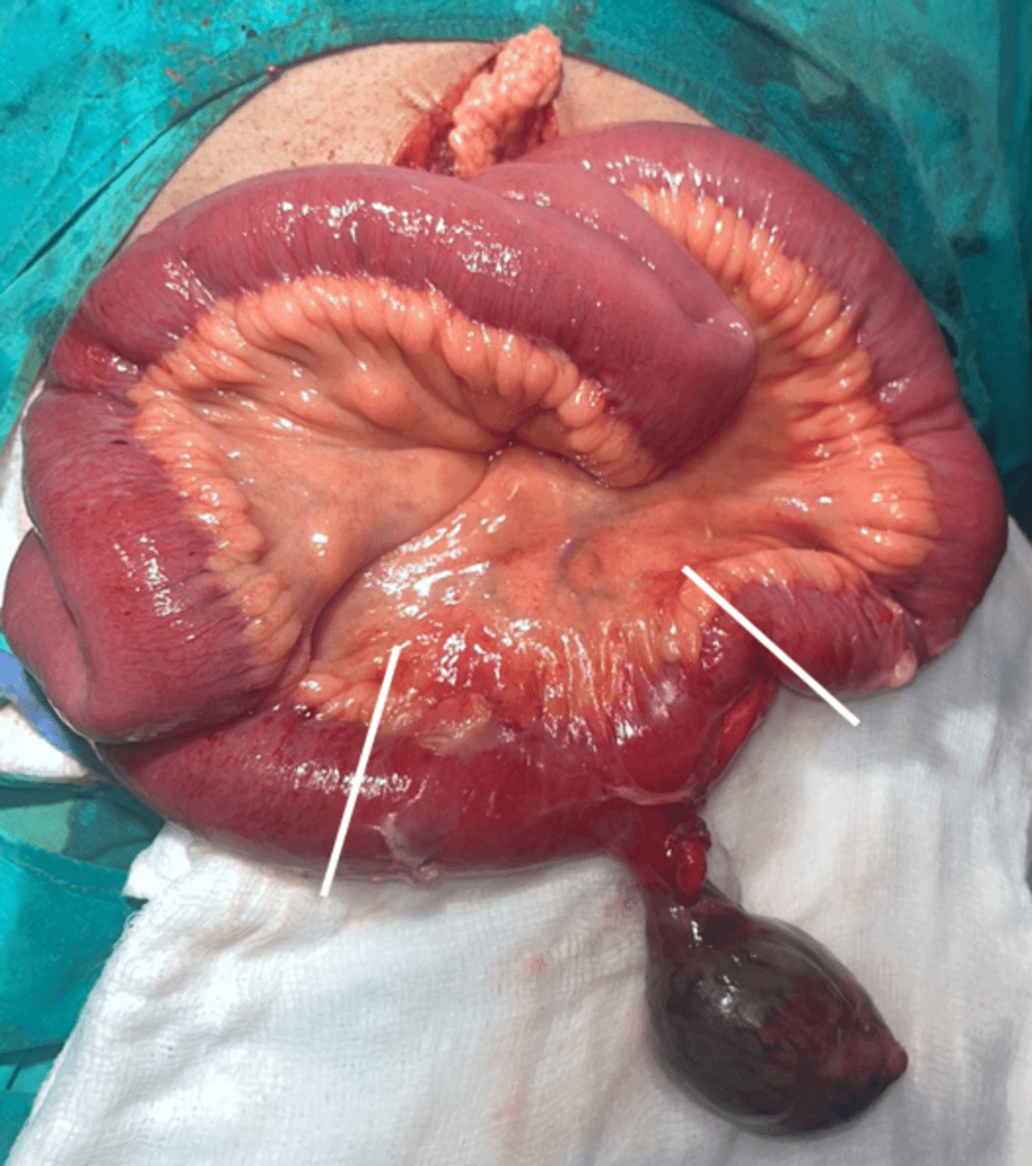
Margins of resection (solid white lines) around the Meckel's diverticulum at the junction of healthy and edematous bowel.

These findings raised the suspicion of acute appendicitis with the possibility of perforation. In view of the hemodynamic instability of the patient, he was resuscitated and immediately prepared for emergency exploratory laparotomy. A lower midline incision was taken, a thorough wash was given, and the bowel was mapped. To our surprise, the appendix was found to be normal. A 7x5x5 cm gangrenous diverticulum was found on the anti-mesenteric border of the ileum, axially twisted upon its stalk approximately 60 cm proximal to the ileocaecal junction (Figure 2). A diagnosis of torsion of Meckel’s diverticulum was made. Taking into account the nearby oedematous bowel, approximately 5 cm of the small intestine was resected on either side of the diverticulum and anastomosed with Vicryl 2-0 (Ethicon, Inc., Raritan, New Jersey, United States) (Figure 3). An abdominal drain was placed in the pelvis, and the abdomen closed.

**Figure 4:**
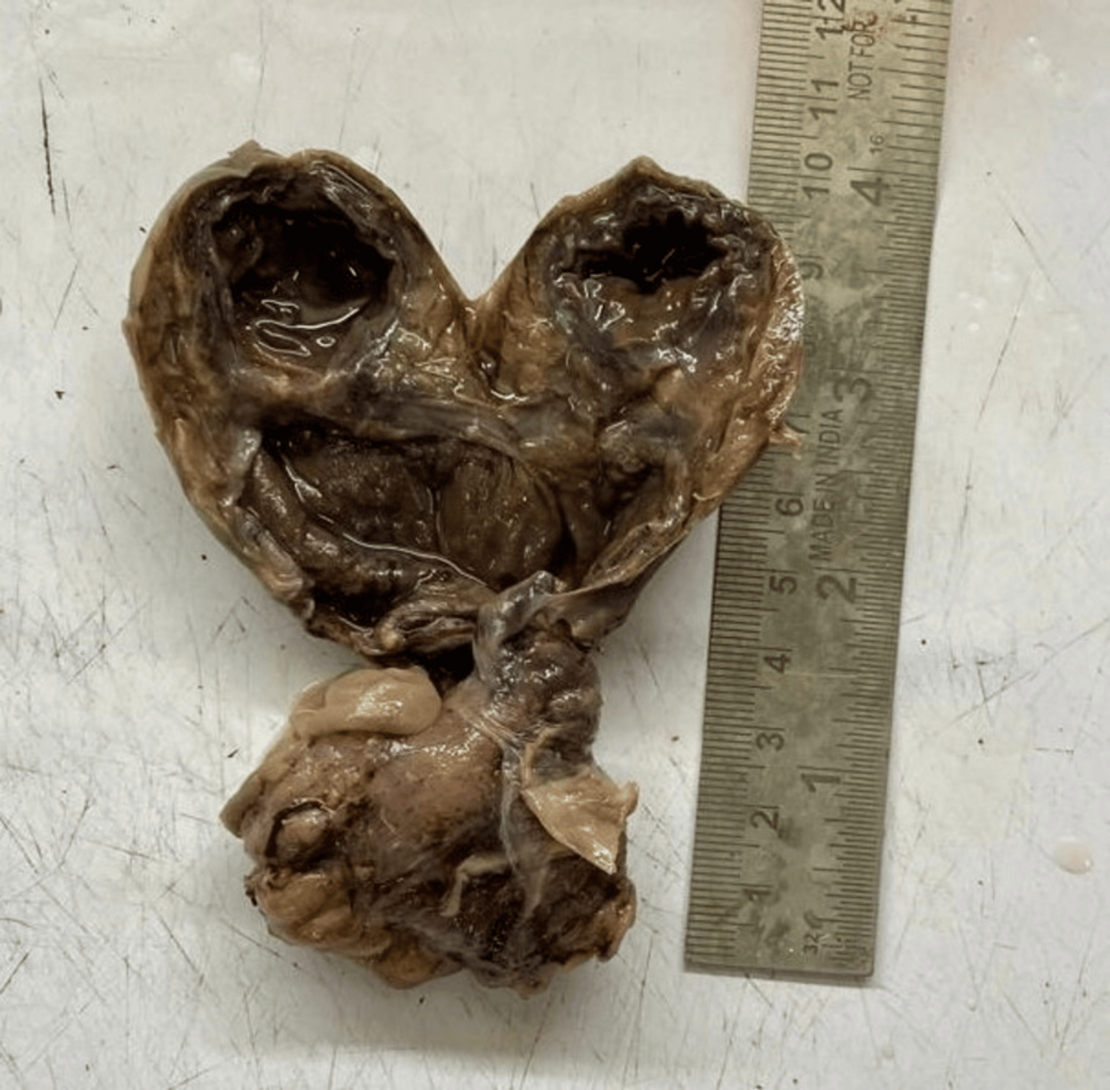
Excised specimen of gangrenous giant Meckel's diverticulum on pathological examination.

**Figure 5:**
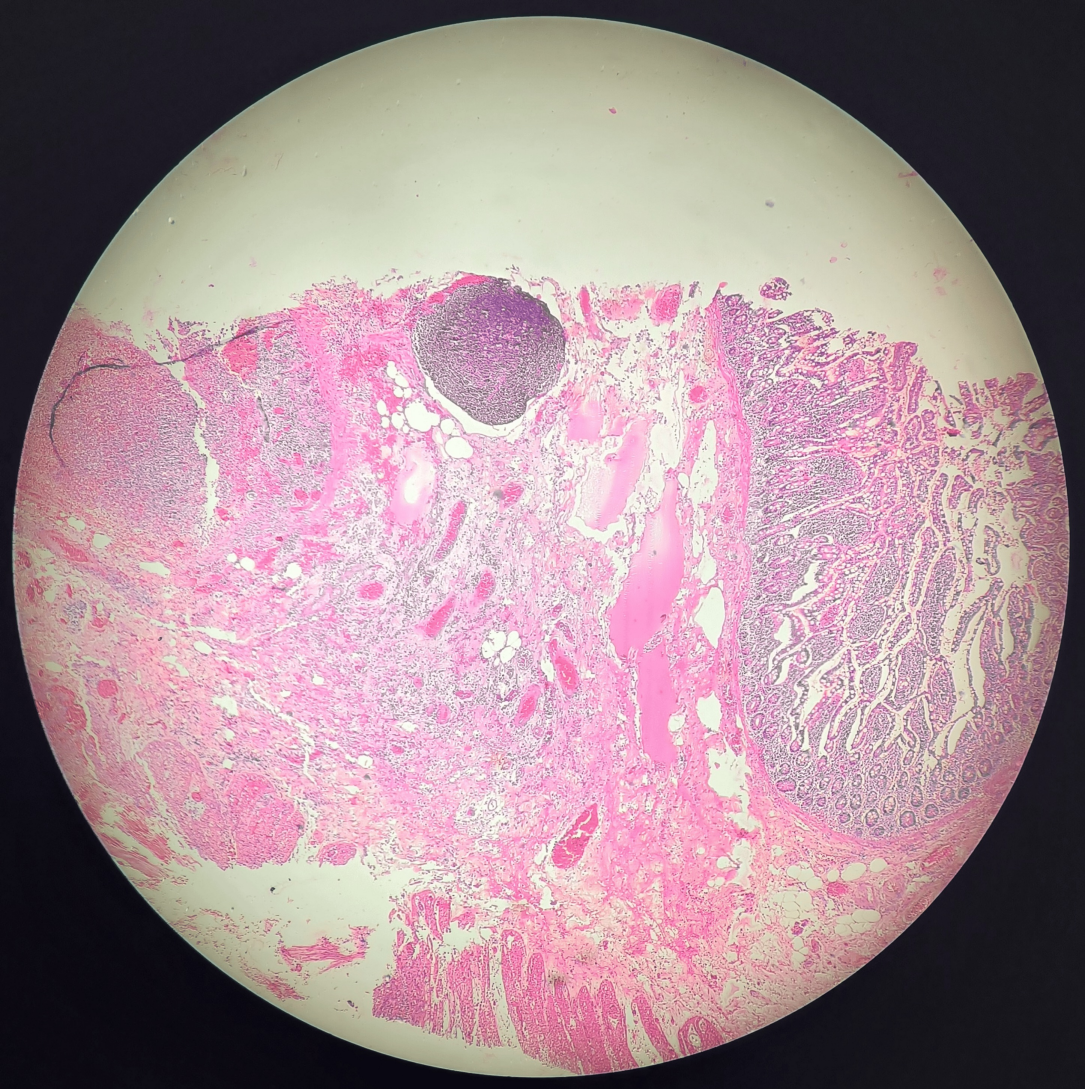
Evidence of all three layers of the gastrointestinal tract is noted on histopathological examination, proving that it is a true diverticulum.

The patient had an uneventful postoperative course and was discharged home smoothly seven days later on a full diet. He remains asymptomatic on follow-up. The specimen that was sent for histopathological examination supported the diagnosis. There was no ectopic tissue present (Figure 4 and Figure 5).

## Discussion

Meckel's diverticulum is the most common congenital malformation of the GIT and was discovered by Johann Friedrich Meckel in 1809 [1,2]. Following the rule of 2s, it has an incidence of 2%, is usually 2 inches long and 2 cm in diameter, and is found 2 feet proximal to the ileocecal junction. Additionally, only 2-4% of patients are symptomatic, and it is twice as common in men [2,3]. Approximately 50% of cases show heterotopic tissue of predominantly two kinds: gastric (60-85% of these) and sometimes pancreatic (5-16%). Occasionally, colonic or endometrial tissue has been found [3,8].

During embryological development, the vitelline (or omphalomesenteric) duct connects the yolk sac to the lumen of the developing GIT and obliterates by the fifth to seventh week of fetal development [4]. Failure of complete involution can result in a persistent vitelline duct, vitelline cysts, vitelline sinuses, or a vitelline band. Persistence on the gut side results in a Meckel’s diverticulum. This makes it a true diverticulum, comprising all the layers of the gut. The pluripotent nature of the vitelline cell line predisposes to the presence of heterotopic tissue [3,9].

Less than 0.5% of vitelline anomalies are found to be giant Meckel's diverticula, where the diverticulum is greater than 5 cm in length; 80% of cases are asymptomatic, but the risk of developing a complication is significantly higher, going up to 6.4% compared to the 2% risk with a smaller Meckel’s [10].

Yamaguchi and colleagues, in a study of 600 patients, showed that the distance from the ileocecal junction varies with age. It is at an average distance of 34 cm for children under two years and around 67 cm on average for adults [11].

Meckel's diverticula can cause symptoms or remain clinically silent. Of the patients, 2-4% present with lower gastrointestinal bleeding (melena) or acute abdomen related to intestinal obstruction, Meckel's diverticulitis, or perforation. Of these, 25-50% of symptomatic patients present in childhood. Rarely, Littre’s hernia or tumors may be found. Axial torsion of the diverticulum followed by gangrene, is one of the rarest complications that can occur [4,12].

Meckel's diverticula may contain heterotopic gastric tissue, which secretes hydrochloric acid, causing ulceration of the small bowel just downstream from the diverticulum, resulting in painless bleeding. Children often present with dark red (currant jelly) or maroon stools, while adults present with melena. It accounts for more than half the cases of lower gastrointestinal bleeding in children younger than two years of age. Resultant anemia may be present [7,12].

Abdominal symptoms are caused by complications of Meckel's diverticula such as intestinal obstruction, diverticulitis, or perforation. In children, intestinal obstruction may result from intussusception or volvulus of the diverticulum, which presents non-specifically, with abdominal distention, vomiting, and constipation. Rarely, torsion, herniation, or inflammation of the Meckel's diverticulum may be seen. Meckel's diverticulitis, similar to acute appendicitis, is thought to be due to obstruction of the diverticular lumen. This may be secondary to an enterolith, food, or sometimes a parasite or tumor. This can lead to complications and cause perforation and peritonitis [12]. Abdominal tenderness is said to be more toward the midline as compared to appendicitis, but the position of the diverticulum can vary; thus, the location of pain and tenderness is not specific. Perforation of Meckel's diverticulum will manifest with peritonitis, while an abscess related to Meckel's diverticulum may produce a palpable mass.

Although usually discovered incidentally, a routine contrast-enhanced computed tomography of the abdomen and pelvis may show evidence of inflammation or obstruction at the diverticulum. Other methods include a Meckel scan, tagged red blood cell scan in patients with active bleeding, and diagnostic laparoscopy. The Meckel scan is a radionuclide study that uses technetium-99m, which is absorbed by the ectopic gastric mucosa (if present in the diverticulum) allowing it to be visualized [13,14].

The treatment of symptomatic Meckel diverticulum is surgical excision. For incidentally discovered cases, surgery and observation are both reasonable options and must be decided on a case-to-case basis [3].

Khan et al. proposed that various management options are feasible, including diverticulectomy, wedge resection, and surgical resection [15]. These may be via laparoscopic or open (laparotomy) approaches. The choice of procedure is based on factors such as the presence of ectopic tissue, its location, the width of the diverticular base, and whether the base is healthy. In conjunction with this, and in view of the surrounding edematous bowel, we chose to perform a limited resection and anastomosis to ensure any basal heterotopic tissue (if present) was also excised, ensuring that future complications related to the diverticulum would not arise.

## Conclusions

It is important to bear in mind Meckel’s diverticulum and its complications as an important differential of acute abdomen. Additionally, every general surgeon must remember routine bowel mapping during procedures such as laparoscopic or open appendicectomy, exploratory laparotomy, and diagnostic laparoscopy. This simple practice is a must to minimize the potential serious morbidity that is associated with complications related to Meckel's diverticulum.
